# 4-(4-Methoxy­phen­yl)-3-methyl-1,6-di­oxa-2,8-diaza-*s*-indacen-5(7*H*)-one

**DOI:** 10.1107/S1600536809013373

**Published:** 2009-04-18

**Authors:** Li-Xin Zhang, Xiao-Hong Zhang, Shu Yan

**Affiliations:** a College of Chemistry and Chemical Engineering, Xuzhou Normal University, Xuzhou 221116, People’s Republic of China

## Abstract

In the mol­ecule of the title compound, C_16_H_12_N_2_O_4_, the pyridine ring is oriented at the same dihedral angle of 2.92 (3)° with respect to the furan and isoxazole rings, while the dihedral angle between furan and isoxazole rings is 1.34 (3)°. The dihedral angle between the benzene and pyridine rings is 53.23 (3)°. In the crystal structure, inter­molecular C—H⋯O inter­actions link the mol­ecules into chains. Weak π–π contacts between isoxazole and benzene rings [centroid–centroid distance = 3.969 (3) Å] may further stabilize the structure.

## Related literature

For general background to isoxazoles, see: Pinho & Teresa (2005 ▶); Shin *et al.* (2005 ▶); Tatee *et al.* (1987 ▶). For a related structure, see: Chande *et al.* (2005 ▶). For bond-length data, see: Allen *et al.* (1987 ▶).
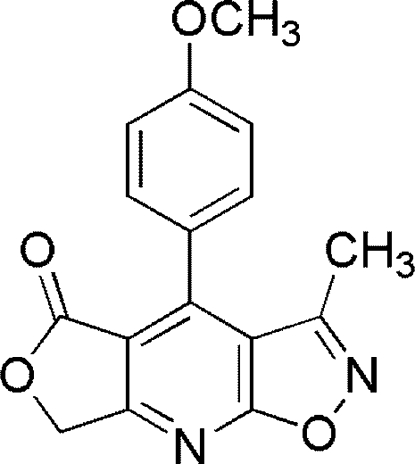


## Experimental

### 

#### Crystal data

C_16_H_12_N_2_O_4_
*M* *_r_* = 296.28
Monoclinic, 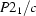

*a* = 13.8513 (16) Å
*b* = 7.6116 (11) Å
*c* = 12.6732 (15) Å
β = 95.592 (1)°
*V* = 1329.8 (3) Å^3^
*Z* = 4
Mo *K*α radiation
μ = 0.11 mm^−1^
*T* = 298 K
0.14 × 0.11 × 0.05 mm

#### Data collection

Bruker SMART CCD area-detector diffractometer
Absorption correction: multi-scan (*SADABS*; Sheldrick, 1996 ▶) *T*_min_ = 0.985, *T*_max_ = 0.995
6625 measured reflections
2333 independent reflections
1267 reflections with *I* > 2σ(*I*)
*R*_int_ = 0.085

#### Refinement

*R*[*F* ^2^ > 2σ(*F* ^2^)] = 0.058
*wR*(*F* ^2^) = 0.093
*S* = 1.03
2333 reflections
201 parameters
H-atom parameters constrained
Δρ_max_ = 0.14 e Å^−3^
Δρ_min_ = −0.19 e Å^−3^

### 

Data collection: *SMART* (Bruker, 1998 ▶); cell refinement: *SAINT* (Bruker, 1998 ▶); data reduction: *SAINT*; program(s) used to solve structure: *SHELXS97* (Sheldrick, 2008 ▶); program(s) used to refine structure: *SHELXL97* (Sheldrick, 2008 ▶); molecular graphics: *ORTEP-3 for Windows* (Farrugia, 1997 ▶) and *PLATON* (Spek, 2009 ▶); software used to prepare material for publication: *SHELXL97*.

## Supplementary Material

Crystal structure: contains datablocks global, I. DOI: 10.1107/S1600536809013373/hk2664sup1.cif

Structure factors: contains datablocks I. DOI: 10.1107/S1600536809013373/hk2664Isup2.hkl

Additional supplementary materials:  crystallographic information; 3D view; checkCIF report

## Figures and Tables

**Table 1:** Hydrogen-bond geometry (Å, °)

*D*—H⋯*A*	*D*—H	H⋯*A*	*D*⋯*A*	*D*—H⋯*A*
C2—H2*B*⋯O2^i^	0.97	2.39	3.215 (3)	143

Symmetry code: (i) 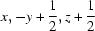.

**Fig. 1.**
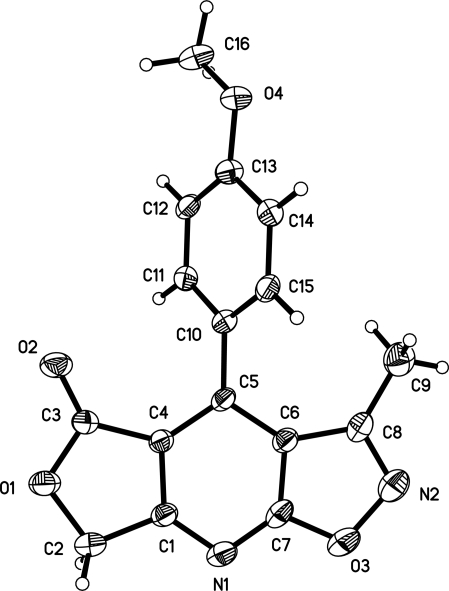
The molecular structure of the title molecule, with the atom-numbering scheme. Displacement ellipsoids are drawn at the 30% probability level.

**Fig. 2.**
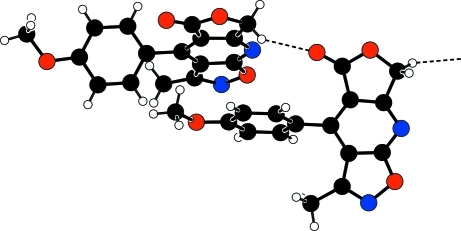
A partial packing diagram of the title compound. Hydrogen bonds are shown as dashed lines.

## supplementary crystallographic information

### Comment

Isoxazole is one of the important heterocyclic units, which has been widely used as a key building block for pharmaceutical agents. Its derivatives are endowed with many pharmacological properties, such as hypoglycemic, analgesic, anti-inflammatory, anti-bacterial, anti-cancer and anti-HIV activities (Shin *et al.*,  2005). Besides, they also have agrochemical properties including herbicidal and soil fungicidal activities, thus they have been used as pesticides and insecticides (Pinho & Teresa, 2005). Among the derivatives of isoxazole, isoxazolopyridine has evoked people's interest and concern, since it showed muscle relaxant, anticonvulsant and CNS depressant activities (Tatee *et al.*,  1987). To the best of our knowledge, modification and synthesis of polycyclic-fused isoxazolopyridine have never been reported. Thus, synthesis of structurally diverse isoxazole-based (Chande *et al.*,  2005) small molecules is of great significance. We report herein the crystal structure of the title compound.

In the molecule of the title compound (Fig. 1), the bond lengths (Allen *et al.*,  1987) and angles are within normal ranges. Rings A (O1/C1-C4), B (O3/N2/C6-C8), C (N1/C1/C4-C7) and D (C10-C15) are, of course, planar, and they are oriented at dihedral angles of A/B = 1.34 (3), A/C = 2.92 (3), A/D = 56.13 (4), B/C = 2.92 (3), B/D = 55.97 (4) and C/D = 53.23 (3) °.

In the crystal structure, intermolecular C-H···O interactions (Table 1) link the molecules into chains (Fig. 2), in which they may be effective in the stabilization of the structure. The π–π contact between the isoxazole and phenyl rings, Cg2—Cg4^i^ [symmetry code: (i) x, 3/2 - y, z + 1/2, where Cg2 and Cg4 are centroids of the rings B (O3/N2/C6-C8) and D (C10-C15), respectively] may further stabilize the structure, with centroid-centroid distance of 3.969 (3) Å.

### Experimental

The title compound was prepared by the reaction of 4-methoxybenzaldehyde (1 mmol), tetronic acid (1 mmol) and 3-methylisoxazol-5-amine (1 mmol) in water (2 ml). Crystals suitable for X-ray analysis were obtained by slow evaporation of an aqueous ethanol solution (95%) (yield; 91%, m.p. 504-506 K). IR (cm^-1^): 1759; ^1^H NMR (DMSO-d_6_): 7.56 (d, 2H, J = 8.8 Hz, ArH), 7.12 (d, 2H, J = 8.8 Hz, ArH), 5.49 (s, 2H, CH2), 3.87 (s, 3H, OCH3), 2.16 (s, 3H, CH3); ^13^C NMR (DMSO-d_6_): 171.84, 170.16, 167.00, 160.71, 157.07, 149.38, 131.54, 121.73, 113.40, 113.30, 112.89, 68.72, 55.30, 12.86.

### Refinement

H atoms were positioned geometrically, with C-H = 0.93, 0.97 and 0.96 Å for aromatic, methylene and methyl H, respectively, and constrained to ride on their parent atoms, with U_iso_(H) = xU_eq_(C), where x = 1.5 for methyl H and x = 1.2 for all other H atoms.

### Crystal data

**Table:**

C_16_H_12_N_2_O_4_	*F*(000) = 616
*M**_r_* = 296.28	*D*_x_ = 1.480 Mg m^−^^3^
Monoclinic, *P*2_1_/*c*	Melting point = 504–506 K
Hall symbol: -P 2ybc	Mo *K*α radiation, λ = 0.71073 Å
*a* = 13.8513 (16) Å	Cell parameters from 1263 reflections
*b* = 7.6116 (11) Å	θ = 3.0–27.5°
*c* = 12.6732 (15) Å	µ = 0.11 mm^−^^1^
β = 95.592 (1)°	*T* = 298 K
*V* = 1329.8 (3) Å^3^	Block, colorless
*Z* = 4	0.14 × 0.11 × 0.05 mm

### Data collection

**Table:**

Bruker SMART CCD area-detector diffractometer	2333 independent reflections
Radiation source: fine-focus sealed tube	1267 reflections with *I* > 2σ(*I*)
graphite	*R*_int_ = 0.085
φ and ω scans	θ_max_ = 25.0°, θ_min_ = 1.5°
Absorption correction: multi-scan (*SADABS*; Sheldrick, 1996)	*h* = −16→16
*T*_min_ = 0.985, *T*_max_ = 0.995	*k* = −5→9
6625 measured reflections	*l* = −15→15

### Refinement

**Table:**

Refinement on *F*^2^	Secondary atom site location: difference Fourier map
Least-squares matrix: full	Hydrogen site location: inferred from neighbouring sites
*R*[*F*^2^ > 2σ(*F*^2^)] = 0.058	H-atom parameters constrained
*wR*(*F*^2^) = 0.093	*w* = 1/[σ^2^(*F*_o_^2^) + (0.0242*P*)^2^] where *P* = (*F*_o_^2^ + 2*F*_c_^2^)/3
*S* = 1.02	(Δ/σ)_max_ < 0.001
2333 reflections	Δρ_max_ = 0.14 e Å^−^^3^
201 parameters	Δρ_min_ = −0.19 e Å^−^^3^
Primary atom site location: structure-invariant direct methods	

### Special details

**Table:**

Geometry. All e.s.d.'s (except the e.s.d. in the dihedral angle between two l.s. planes) are estimated using the full covariance matrix. The cell e.s.d.'s are taken into account individually in the estimation of e.s.d.'s in distances, angles and torsion angles; correlations between e.s.d.'s in cell parameters are only used when they are defined by crystal symmetry. An approximate (isotropic) treatment of cell e.s.d.'s is used for estimating e.s.d.'s involving l.s. planes.
Refinement. Refinement of *F*^2^ against ALL reflections. The weighted *R*-factor *wR* and goodness of fit *S* are based on *F*^2^, conventional *R*-factors *R* are based on *F*, with *F* set to zero for negative *F*^2^. The threshold expression of *F*^2^ > σ(*F*^2^) is used only for calculating *R*-factors(gt) *etc*. and is not relevant to the choice of reflections for refinement. *R*-factors based on *F*^2^ are statistically about twice as large as those based on *F*, and *R*- factors based on ALL data will be even larger.

### Fractional atomic coordinates and isotropic or equivalent isotropic displacement parameters (Å^2^)

**Table:**

	*x*	*y*	*z*	*U*_iso_*/*U*_eq_	
N1	0.19564 (17)	−0.0001 (3)	0.93775 (17)	0.0496 (6)	
N2	0.42384 (17)	−0.1679 (3)	0.8929 (2)	0.0646 (7)	
O1	−0.00728 (12)	0.1886 (3)	0.77882 (13)	0.0530 (5)	
O2	0.03096 (12)	0.1945 (3)	0.61265 (14)	0.0561 (5)	
O3	0.35098 (14)	−0.1183 (3)	0.95948 (14)	0.0625 (6)	
O4	0.30666 (13)	0.0742 (2)	0.28784 (14)	0.0553 (6)	
C1	0.1314 (2)	0.0595 (3)	0.8621 (2)	0.0413 (7)	
C2	0.03488 (19)	0.1313 (4)	0.8820 (2)	0.0545 (8)	
H2A	−0.0049	0.0415	0.9106	0.065*	
H2B	0.0418	0.2291	0.9312	0.065*	
C3	0.05378 (18)	0.1546 (4)	0.7033 (2)	0.0431 (7)	
C4	0.14180 (18)	0.0708 (3)	0.75423 (18)	0.0371 (6)	
C5	0.22780 (17)	0.0190 (3)	0.71290 (19)	0.0364 (6)	
C6	0.29723 (19)	−0.0463 (3)	0.7918 (2)	0.0411 (7)	
C7	0.2760 (2)	−0.0493 (4)	0.8971 (2)	0.0461 (7)	
C8	0.3921 (2)	−0.1270 (4)	0.7960 (2)	0.0496 (7)	
C9	0.45235 (19)	−0.1739 (4)	0.7089 (2)	0.0653 (9)	
H9A	0.4837	−0.0703	0.6857	0.098*	
H9B	0.4117	−0.2232	0.6507	0.098*	
H9C	0.5006	−0.2584	0.7344	0.098*	
C10	0.24536 (17)	0.0332 (3)	0.60100 (18)	0.0366 (6)	
C11	0.18199 (17)	−0.0388 (3)	0.52099 (19)	0.0410 (7)	
H11	0.1263	−0.0955	0.5385	0.049*	
C12	0.19987 (17)	−0.0281 (3)	0.41641 (19)	0.0424 (7)	
H12	0.1567	−0.0784	0.3642	0.051*	
C13	0.28166 (18)	0.0570 (4)	0.3886 (2)	0.0411 (7)	
C14	0.34544 (18)	0.1327 (3)	0.4676 (2)	0.0438 (7)	
H14	0.4002	0.1921	0.4498	0.053*	
C15	0.32724 (17)	0.1194 (3)	0.5715 (2)	0.0420 (7)	
H15	0.3706	0.1691	0.6237	0.050*	
C16	0.2431 (2)	0.0018 (4)	0.2031 (2)	0.0576 (8)	
H16A	0.2355	−0.1219	0.2145	0.086*	
H16B	0.2703	0.0203	0.1371	0.086*	
H16C	0.1810	0.0584	0.2008	0.086*	

### Atomic displacement parameters (Å^2^)

**Table:**

	*U*^11^	*U*^22^	*U*^33^	*U*^12^	*U*^13^	*U*^23^
N1	0.0622 (16)	0.0493 (17)	0.0355 (14)	−0.0014 (14)	−0.0045 (12)	0.0028 (12)
N2	0.0610 (16)	0.0695 (19)	0.0598 (18)	0.0076 (15)	−0.0117 (14)	0.0066 (14)
O1	0.0524 (11)	0.0708 (14)	0.0364 (12)	0.0069 (11)	0.0064 (9)	0.0018 (10)
O2	0.0512 (11)	0.0798 (15)	0.0365 (12)	0.0089 (11)	0.0012 (9)	0.0128 (11)
O3	0.0690 (13)	0.0704 (16)	0.0447 (13)	0.0046 (12)	−0.0123 (11)	0.0081 (11)
O4	0.0597 (12)	0.0746 (15)	0.0319 (12)	−0.0042 (11)	0.0065 (10)	−0.0003 (10)
C1	0.0515 (16)	0.0396 (18)	0.0320 (16)	−0.0066 (14)	−0.0008 (13)	−0.0008 (13)
C2	0.0623 (19)	0.067 (2)	0.0340 (17)	−0.0022 (17)	0.0060 (14)	−0.0004 (15)
C3	0.0476 (17)	0.048 (2)	0.0345 (17)	−0.0057 (15)	0.0068 (14)	−0.0007 (14)
C4	0.0417 (15)	0.0376 (17)	0.0311 (16)	−0.0049 (13)	−0.0001 (12)	0.0018 (12)
C5	0.0430 (15)	0.0349 (17)	0.0294 (15)	−0.0061 (13)	−0.0056 (12)	−0.0006 (12)
C6	0.0458 (16)	0.0413 (18)	0.0351 (17)	−0.0052 (14)	−0.0020 (13)	−0.0021 (13)
C7	0.0537 (18)	0.0376 (19)	0.0431 (19)	−0.0019 (15)	−0.0154 (15)	0.0031 (14)
C8	0.0483 (17)	0.049 (2)	0.0489 (19)	−0.0036 (16)	−0.0102 (14)	−0.0005 (15)
C9	0.0561 (18)	0.069 (2)	0.069 (2)	0.0130 (18)	−0.0001 (16)	0.0028 (17)
C10	0.0384 (15)	0.0395 (18)	0.0310 (16)	0.0010 (13)	−0.0012 (12)	0.0006 (12)
C11	0.0371 (15)	0.0471 (19)	0.0385 (18)	−0.0041 (13)	0.0015 (12)	0.0026 (13)
C12	0.0411 (16)	0.050 (2)	0.0339 (17)	−0.0022 (15)	−0.0058 (13)	−0.0066 (13)
C13	0.0441 (16)	0.0436 (19)	0.0355 (17)	0.0061 (14)	0.0030 (13)	−0.0004 (13)
C14	0.0389 (15)	0.051 (2)	0.0418 (18)	−0.0034 (14)	0.0049 (13)	0.0020 (14)
C15	0.0380 (15)	0.0470 (19)	0.0393 (17)	−0.0039 (14)	−0.0052 (12)	−0.0028 (14)
C16	0.080 (2)	0.057 (2)	0.0342 (17)	0.0050 (18)	−0.0010 (15)	−0.0030 (15)

### Geometric parameters (Å, °)

**Table:**

N1—C1	1.324 (3)	C6—C8	1.446 (3)
N1—C7	1.325 (3)	C8—C9	1.490 (4)
N2—C8	1.302 (3)	C9—H9A	0.9600
N2—O3	1.428 (3)	C9—H9B	0.9600
O1—C3	1.363 (3)	C9—H9C	0.9600
O1—C2	1.446 (3)	C10—C11	1.388 (3)
O2—C3	1.200 (3)	C10—C15	1.393 (3)
O3—C7	1.349 (3)	C11—C12	1.374 (3)
O4—C13	1.361 (3)	C11—H11	0.9300
O4—C16	1.431 (3)	C12—C13	1.380 (3)
C1—C4	1.391 (3)	C12—H12	0.9300
C1—C2	1.488 (3)	C13—C14	1.394 (3)
C2—H2A	0.9700	C14—C15	1.368 (3)
C2—H2B	0.9700	C14—H14	0.9300
C3—C4	1.469 (3)	C15—H15	0.9300
C4—C5	1.404 (3)	C16—H16A	0.9600
C5—C6	1.409 (3)	C16—H16B	0.9600
C5—C10	1.466 (3)	C16—H16C	0.9600
C6—C7	1.395 (3)		
			
C1—N1—C7	110.3 (2)	C6—C8—C9	130.3 (3)
C8—N2—O3	107.5 (2)	C8—C9—H9A	109.5
C3—O1—C2	110.7 (2)	C8—C9—H9B	109.5
C7—O3—N2	107.8 (2)	H9A—C9—H9B	109.5
C13—O4—C16	118.2 (2)	C8—C9—H9C	109.5
N1—C1—C4	127.3 (3)	H9A—C9—H9C	109.5
N1—C1—C2	123.7 (2)	H9B—C9—H9C	109.5
C4—C1—C2	109.0 (2)	C11—C10—C15	117.6 (2)
O1—C2—C1	104.4 (2)	C11—C10—C5	121.7 (2)
O1—C2—H2A	110.9	C15—C10—C5	120.7 (2)
C1—C2—H2A	110.9	C12—C11—C10	121.4 (2)
O1—C2—H2B	110.9	C12—C11—H11	119.3
C1—C2—H2B	110.9	C10—C11—H11	119.3
H2A—C2—H2B	108.9	C11—C12—C13	120.2 (2)
O2—C3—O1	120.0 (2)	C11—C12—H12	119.9
O2—C3—C4	131.4 (2)	C13—C12—H12	119.9
O1—C3—C4	108.6 (2)	O4—C13—C12	125.1 (2)
C1—C4—C5	121.5 (2)	O4—C13—C14	115.6 (2)
C1—C4—C3	107.3 (2)	C12—C13—C14	119.3 (2)
C5—C4—C3	131.0 (2)	C15—C14—C13	119.8 (2)
C4—C5—C6	112.3 (2)	C15—C14—H14	120.1
C4—C5—C10	124.6 (2)	C13—C14—H14	120.1
C6—C5—C10	123.1 (2)	C14—C15—C10	121.7 (2)
C7—C6—C5	119.5 (3)	C14—C15—H15	119.2
C7—C6—C8	103.5 (2)	C10—C15—H15	119.2
C5—C6—C8	136.9 (3)	O4—C16—H16A	109.5
N1—C7—O3	120.7 (3)	O4—C16—H16B	109.5
N1—C7—C6	129.1 (3)	H16A—C16—H16B	109.5
O3—C7—C6	110.2 (3)	O4—C16—H16C	109.5
N2—C8—C6	111.0 (2)	H16A—C16—H16C	109.5
N2—C8—C9	118.6 (3)	H16B—C16—H16C	109.5
			
C8—N2—O3—C7	0.6 (3)	N2—O3—C7—C6	−1.7 (3)
C7—N1—C1—C4	0.6 (4)	C5—C6—C7—N1	1.1 (4)
C7—N1—C1—C2	−177.6 (2)	C8—C6—C7—N1	−176.1 (3)
C3—O1—C2—C1	1.0 (3)	C5—C6—C7—O3	179.2 (2)
N1—C1—C2—O1	176.8 (2)	C8—C6—C7—O3	2.0 (3)
C4—C1—C2—O1	−1.6 (3)	O3—N2—C8—C6	0.7 (3)
C2—O1—C3—O2	−179.5 (2)	O3—N2—C8—C9	−176.8 (2)
C2—O1—C3—C4	0.0 (3)	C7—C6—C8—N2	−1.7 (3)
N1—C1—C4—C5	−1.1 (4)	C5—C6—C8—N2	−178.1 (3)
C2—C1—C4—C5	177.3 (2)	C7—C6—C8—C9	175.4 (3)
N1—C1—C4—C3	−176.8 (3)	C5—C6—C8—C9	−1.0 (5)
C2—C1—C4—C3	1.7 (3)	C4—C5—C10—C11	−53.8 (4)
O2—C3—C4—C1	178.3 (3)	C6—C5—C10—C11	127.3 (3)
O1—C3—C4—C1	−1.1 (3)	C4—C5—C10—C15	126.5 (3)
O2—C3—C4—C5	3.2 (5)	C6—C5—C10—C15	−52.4 (4)
O1—C3—C4—C5	−176.1 (2)	C15—C10—C11—C12	1.0 (4)
C1—C4—C5—C6	1.4 (3)	C5—C10—C11—C12	−178.7 (2)
C3—C4—C5—C6	175.9 (3)	C10—C11—C12—C13	−0.7 (4)
C1—C4—C5—C10	−177.6 (2)	C16—O4—C13—C12	1.1 (4)
C3—C4—C5—C10	−3.1 (4)	C16—O4—C13—C14	−179.0 (2)
C4—C5—C6—C7	−1.4 (3)	C11—C12—C13—O4	179.5 (2)
C10—C5—C6—C7	177.7 (2)	C11—C12—C13—C14	−0.4 (4)
C4—C5—C6—C8	174.6 (3)	O4—C13—C14—C15	−178.8 (2)
C10—C5—C6—C8	−6.4 (5)	C12—C13—C14—C15	1.1 (4)
C1—N1—C7—O3	−178.5 (2)	C13—C14—C15—C10	−0.8 (4)
C1—N1—C7—C6	−0.6 (4)	C11—C10—C15—C14	−0.3 (4)
N2—O3—C7—N1	176.5 (2)	C5—C10—C15—C14	179.4 (2)

### Hydrogen-bond geometry (Å, °)

**Table:**

*D*—H···*A*	*D*—H	H···*A*	*D*···*A*	*D*—H···*A*
C2—H2B···O2^i^	0.97	2.39	3.215 (3)	143

Symmetry codes: (i) *x*, −*y*+1/2, *z*+1/2.
